# Characterization of [^99m^Tc]Duramycin as a SPECT Imaging Agent for Early Assessment of Tumor Apoptosis

**DOI:** 10.1007/s11307-015-0852-6

**Published:** 2015-04-21

**Authors:** Filipe Elvas, Christel Vangestel, Sara Rapic, Jeroen Verhaeghe, Brian Gray, Koon Pak, Sigrid Stroobants, Steven Staelens, Leonie wyffels

**Affiliations:** Molecular Imaging Center Antwerp, University of Antwerp, Antwerp, Belgium; Department of Nuclear Medicine, University Hospital Antwerp, Wilrijkstraat 10, 2650 Edegem, Belgium; Molecular Targeting Technologies, Inc., West Chester, PA USA

**Keywords:** ^99m^Tc, Duramycin, Preclinical dosimetry, Small-animal SPECT imaging, Chemotherapy, Apoptosis

## Abstract

**Purpose:**

We investigated the usefulness of [^99m^Tc]duramycin for monitoring early response to cancer therapy in mice, with an eye towards clinical translation.

**Procedures:**

[^99m^Tc]Duramycin was injected in healthy CD1−/− mice to estimate human [^99m^Tc]duramycin radiation dose. [^99m^Tc]Duramycin single-photon emission computed tomography (SPECT) imaging of apoptosis was evaluated in a mouse model of colorectal cancer treated with irinotecan and validated *ex vivo* using autoradiography, cleaved caspase-3, and TdT-mediated dUTP nick-end labeling (TUNEL) histology of the tumors.

**Results:**

The mean effective dose was estimated to be 3.74 × 10^−3^ ± 3.43 × 10^−4^ mSv/MBq for non-purified and 3.19 × 10^−3^ ± 2.16 × 10^−4^ mSv/MBq for purified [^99m^Tc]duramycin. [^99m^Tc]Duramycin uptake *in vivo* following therapy increased significantly in apoptotic irinotecan-treated tumors (*p* = 0.008). Radioactivity in the tumors positively correlated with cleaved caspase-3 (*r* = 0.85, *p* < 0.001) and TUNEL (*r* = 0.92, *p* < 0.001) staining.

**Conclusion:**

[^99m^Tc]Duramycin can be used to detect early chemotherapy-induced tumor cell death, and thus, may be a prospective candidate for clinical SPECT imaging of tumor response to therapy.

**Electronic supplementary material:**

The online version of this article (doi:10.1007/s11307-015-0852-6) contains supplementary material, which is available to authorized users.

## Introduction

Monitoring early tumor response to therapy is a major priority in oncology. Currently, the most commonly used methodology to evaluate the effectiveness of a given therapeutic regimen is still based on morphological and volumetric criteria (*e.g.*, RECIST) [1]. However, these methods are not sensitive enough to assess early effects of therapy. Molecular imaging can offer clinicians a direct and specific tool to follow-up response to cancer therapy, allowing changing therapy early on in non-responding patients, avoiding unnecessary toxicity and increasing treatment effectiveness.

Apoptosis is a highly regulated process that plays a vital role in the pathogenesis of cancer [2]. The ability to evade apoptosis is an established hallmark of cancer [3] and most cancer therapies such as chemotherapy, radiotherapy, or targeted therapies have a pro-apoptotic [4] but also a necrotic effect [5, 6]. Early in the apoptotic cascade, there is a redistribution of cell membrane aminophospholipids, such as phosphatidylethanolamine (PE) and phosphatidylserine (PS), normally present in the inner leaflet of the cell membrane. PE, the second most abundant phospholipid in the membrane bilayer (15–25 %), becomes early accessible during apoptosis due to exposure to the outer leaflet of the cell membrane and during necrosis due to disruption of the cell membrane [7]. Since anti-cancer therapy can result in necrosis and late apoptotic cells can also become necrotic, imaging cell death in all its forms is important from a clinical point of view. To date, several positron emission tomography (PET) and single-photon emission computed tomography (SPECT) cell death imaging agents have already been explored, including radiolabeled annexin-V [8–10], C2A domain of synaptotagmin-I [11], Zn^2+^-DPA [12], and caspase inhibitors [13], but they have all failed to reach clinical usefulness. The most studied one to date is PS-targeting [^99m^Tc]annexin-V. Although this tracer has been used in many studies as the prototypical cell death-targeting imaging agent, it has several limitations, most notably its slow clearance due to its large protein structure. This radiotracer showed a high liver and kidney uptake, thus increasing the background noise from non-targeted tissues. PS externalization is not specific for apoptosis, occurring also in activated macrophages, which are known to be present in tumors after therapy [14]. Furthermore, it is known that PS-expressing cells can be rapidly cleared by macrophages before the radiotracer can target those cells. These findings suggest that [^99m^Tc]annexin-V is unspecific and cannot be exclusively considered a cell death marker. There currently is a need for novel, more selective, and specific small-molecule cell death imaging agents with high stability *in vivo*. Ideally, these agents should have avid target uptake, with high selectivity for dead cells, favorable *in vivo* distribution, with rapid blood clearance and low hepatic background, and be easily integrated in the clinical practice. In this way, PE provides a molecular biomarker for the detection of cell death (apoptosis and necrosis) and has become a target for radiotracer development.

Duramycin is a 19-amino acid peptide that recognizes both apoptotic and necrotic cells by binding to PE with high affinity (*K*_d_ ∼ 5 nM) and specificity [15]. The low molecular weight (MW) of duramycin (∼2 kDa) confers this tracer with favorable pharmacokinetics and biodistribution properties for *in vivo* imaging of apoptosis [16]. [^99m^Tc]Duramycin has been successfully used as a probe for the imaging of cell death in animal models of cerebral [17] and myocardial [18, 19] ischemia–reperfusion, accumulating in tissue sites of injury where there is high apoptotic activity. Additionally, this probe was also effectively used to assess tissue damage after whole-body irradiation [20]. A combination of high affinity, superior *in vivo* performance and the use of a radioisotope (Tc-99m) already implemented in the clinical routine in most hospitals makes [^99m^Tc]duramycin a notable candidate for oncology imaging applications.

We therefore hypothesized that [^99m^Tc]duramycin could potentially be used to quantify treatment-induced tumor cell death. Based on this hypothesis, we evaluated the use of [^99m^Tc]duramycin for imaging of chemotherapy-induced tumor cell death in tumor-bearing mice. A kit formulation was previously developed by Zhao and colleagues to obtain injection-ready [^99m^Tc]duramycin [18]. Since in preliminary studies we found suboptimal purity of kit prepared [^99m^Tc]duramycin, we have first assessed the impact of radiotracer purification on the biodistribution and dosimetry profile. This study will be of value for the future use and possible clinical translation of [^99m^Tc]duramycin and for the development of second generation kit formulations.

## Materials and Methods

### Radiolabeling of Duramycin

A single-step kit formulation (MTTI, USA) composed of 15 μg hydrazinonicotinamide- (HYNIC) duramycin, 30 mg tricine, 9.5 mg trisodium triphenylphosphine-3,3′,3″-trisulfonate, and 6.25 μg SnCl_2_ was used for Tc-99m labeling. Approximately, 1,480 MBq [^99m^Tc]pertechnetate in 500 μl of saline was added to the kit and heated at 80 °C for 20 min. The radiopharmaceutical was used without or with purification. In the last case, [^99m^Tc]duramycin obtained from the kit was purified after radiolabeling using the following high-performance liquid chromatography (HPLC) method. For reverse phase (RP) HPLC, a C_18_ column (Grace Vydac 218TP, 5 μm, 300 Å, 250 × 4.6 mm) was used with a gradient elution of 25 mM NaH_2_PO_4_ pH 6.7 and acetonitrile, a flow rate of 1 ml/min and UV/VIS (*λ* = 215 nm, Shimadzu, Japan) and radio-detection (NaI scintillation detector, Raytest, Germany). After collection of the fraction containing [^99m^Tc]duramycin (retention time of 16 min), solvents were evaporated under N_2_, and the radiopharmaceutical was reconstituted in saline with 5 % ethanol for *in vitro* and *in vivo* use. Radiochemical purity (RCP) was determined by analytical HPLC using the same method used for the purification of the radiotracer.

### Dosimetry and Biodistribution Studies

Experimental procedures and protocols were performed in accordance with European Directive 86/609/EEC Welfare and Treatment of Animals and were approved by the local ethical commission (2013-36, University of Antwerp, Belgium). Healthy immunodeficient CD1−/− nude female mice (Charles River Laboratories, Belgium), weighing 24–29 g were intravenously (i.v.) injected with 37 MBq non-purified (*n* = 3) or purified (*n* = 6) [^99m^Tc]duramycin *via* lateral tail vein catheterization. Immediately at injection, dynamic whole-body images were acquired during 60 min using a SPECT-X-ray computed tomography (CT) scanner (VECTor/CT, MILabs, The Netherlands) equipped with a rat multi-pinhole SPECT collimator. Frames were acquired at 1-min intervals for the first 10 min, followed by 5-min acquisitions for the remaining 50-min period. Thereafter, 30-min static scans (six frames of 5 min) were acquired at 4, 8, and 24 h post-radiotracer injection (p.i.). The energy window was centered at 140 keV photopeak with a 20 % energy window for Tc-99m. For quantitative analysis, SPECT data were reconstructed with ordered subset expectation maximization using 10 iterations of 16 subsets and 1.2-mm^3^ voxel size. Following each μSPECT acquisition, a whole-body high-resolution micro-computed tomography (μCT) scan was performed to obtain anatomical information for segmentation. Throughout the entire SPECT-CT scanning procedure, the mice were kept under isoflurane anesthesia and a constant body temperature was maintained using a heating pad.

Volumes of interest (VOIs) were manually drawn on the μSPECT-CT images using PMOD v3.3 (PMOD Technologies, Switzerland) to delineate the regions with distinct SPECT time-activity pattern: heart, liver, kidney, and urinary bladder, as well as whole-body. The average organ activity per volume was obtained from the co-registered μSPECT images and the non-decay corrected time activity curves (TACs) were extracted for each target organ. In order to relate the scanner units (counts/pixel) to radioactivity concentration (kilobecquerel per cubic centimeter), a scanner calibration factor was estimated by scanning a syringe with a known concentration of Tc-99m. TACs for each organ, as well as the TACs representing the activity in the remainder of the body, were fitted by a single exponential decaying function, which was then integrated to obtain the total number of disintegrations (residence time) within these regions. In order to predict human dosimetry using animal data, interspecies extrapolation is necessary. Thus, the residence times (*R*) were scaled to human values according to the following formula [21]:

$$ \left[{\left(\frac{R}{{\mathrm{g}}_{\mathrm{organ}}}\right)}_{\mathrm{mouse}}\cdot {\left({\mathrm{kg}}_{\mathrm{TBweight}}\right)}_{\mathrm{mouse}}\right]\cdot {\left(\frac{{\mathrm{g}}_{\mathrm{organ}}}{{\mathrm{kg}}_{\mathrm{TBweight}}}\right)}_{\mathrm{human}}={R}_{\mathrm{human}} $$

The human organ and body weight values from the standard adult male phantom taken into account are implemented in the internal dosimetry software (OLINDA/EXM v1.1) [22] and are assumed to be constant across individuals. The body weight and organ masses for the individual mice were obtained post-mortem. For the bladder model, the fraction leaving the body *via* the urinary excretion system (*f*) and the biological half-life of the excretion (*t*_1/2_) were estimated by fitting a first-order linear system to the sum of activity in the bladder and the activity that had been excreted (calculated as total injected activity minus total body activity):

$$ y=f\left(1- \exp \left(\frac{ \ln (2)}{t_{1/2}}t\right)\right) $$

Projected human absorbed doses to various organs and the effective doses (ED) were then computed using the aforementioned dosimetry software (weighting factors from International Commission on Radiological Protection 60) [23], with the human residence times as input, using the standard adult male model and the dynamic voiding bladder model [24] (2-h voiding interval).

After the last static scan (24 h p.i.) blood was collected through cardiac puncture, and the mice were euthanized by cervical dislocation for *ex vivo* biodistribution. The organs and tissues were harvested, weighed and the radioactivity in the samples was measured in an automatic gamma- (*γ*) counter (Wizard^2^ 2480, Perkin Elmer, USA) using an energy window of 140 ± 19 keV. Uptake levels of [^99m^Tc]duramycin are presented as percentage injected dose per gram (%I.D./g).

### *In Vitro* Tumor Cell Uptake of [^99m^Tc]Duramycin

A semi-adherent human colorectal adenocarcinoma cell line (COLO205; firefly luciferase gene 2 transfected; Perkin Elmer, Belgium), was grown as previously described [9]. The cells were maintained in RPMI 1640 medium supplemented with 2 mM l-glutamine, 1 mM sodium pyruvate, 10 % fetal bovine serum and 100 U/ml penicillin plus 100 mg/ml streptomycin (Life Technologies, Belgium) at 37 ° C in a humidified atmosphere containing 5 % CO_2_. The cells were kept in exponential phase by routine passage every 2 to 3 days (split ratio: 1/2–1/6), and then plated into six-well plates (Thermo Scientific, USA) at a density of 5.3 × 10^3^ cells/cm^2^. Two days after, COLO205 cells were treated with three increasing concentrations of irinotecan (0.43, 4.3, and 43 μM; Teva, Belgium) or left untreated (control group) for 40 h (*n* = 6 per group). [^99m^Tc]duramycin (0.19 MBq; purified) was then incubated in each well at 37 ° C in a humidified atmosphere of 5 % CO_2_ for 30 min. Cells were washed and after centrifugation at 150×*g* for 5 min the supernatant was discarded. Finally, cells were resuspended and counted using Muse Count & Viability Assay (Millipore, USA). After subsequent centrifugation, the cell-bound radioactivity in the pellet was determined by automatic *γ*-counting. Radioactivity (CPM) per 10^3^ cells was determined and uptake expressed as a percentage of untreated cells. The levels of cell death (apoptosis and necrosis) were assessed by using Muse Annexin V & Dead Cell Assay (Millipore, USA), and Muse Caspase 3/7 Assay Kit (Millipore, USA).

### *In Vivo* Tumor Uptake of [^99m^Tc]Duramycin

Female nude mice (*n* = 6) were subcutaneously injected with luciferase-transfected COLO205 cells (2 × 10^6^ in 100 μl) in both hind legs, as previously described [8]. Tumor volume was monitored using a digital caliper before drug administration (baseline values) and at consecutive days after the start of therapy. Volumes were calculated according to the formula: (length × width^2^)/2. Two weeks after inoculation, when the tumors reached a volume of approximately 400 mm^3^ (*n* = 12 tumors), mice were treated using chemotherapy or vehicle. Irinotecan was administered by intra-peritoneal (i.p.) injection (80 mg/kg) three times in 1 week every other day (*n* = 3). Vehicle-treated (0.9 % NaCl), tumor-bearing mice were used as controls (*n* = 3). Relative tumor volume was calculated as follows: RTV = (mean tumor volume during treatment)/(mean tumor volume before treatment start). Percentage of tumor growth inhibition was calculated as follows: %TGI = [1 − (Mean RTV of irinotecan group/Mean RTV of vehicle group) × 100]. Tumor growth was also monitored using whole body bioluminescence imaging (BLI) before and after the treatment. Twenty-four hours after the last injection of irinotecan or vehicle COLO205-bearing mice were i.v. injected with purified [^99m^Tc]duramycin (37 MBq). Four hours p.i., mice were positioned in the scanner and static SPECT images were acquired during 30 min (six frames of 5 min) followed by a μCT scan as described above. VOIs were manually drawn around all the tumor dimensions on the obtained CT images using PMOD software. After delineation, tracer uptake was quantified as: [Total radioactivity in the tumor at the time of scan (in kilobecquerel)/Total radioactivity injected (in kilobecquerel)] and normalized to tumor weight (in gram) and body weight (in kilogram) (percentage injected dose per gram per kilogram). Standardized uptake values (SUVs) were used to evaluate [^99m^Tc]duramycin uptake by bone marrow. Three-dimensional regions of interest were drawn on the CT images to delineate the bone marrow in the lumbar vertebrae and the sacrum. SUVs were calculated as follows: SUV = [Measured radioactivity concentration (in kilobecquerel per cubic centimeter)]/[Decay-corrected amount of injected activity (in kilobecquerel)/Body weight (in kilogram) × 1,000]. Mice were sacrificed for *ex vivo* biodistribution and the radioactivity in the tumors and organs was determined by *γ*-counting.

### *Ex Vivo* Validation

Immediately after *γ*-counting, tumors (*n* = 6/treatment) were embedded and snap-frozen in tissue-Tek (OCT compound, VWR, USA). For autoradiography studies, frozen tissue sections (100 μm) were sliced, dried at 37 ° C for 1 h and exposed to phosphor screen plates (Fujifilm, USA) overnight. Plates were imaged in a Phosphor Imager system (FLA7000, GE Healthcare, USA). After undergoing radioactive decay, hematoxylin and eosin staining (H&E) was performed in these tumor sections. In parallel, adjacent frozen tumor sections (10 μm) were used for histological analysis of apoptosis, by immunostaining of cleaved caspase-3 (CC3, Cell Signaling, USA) and TdT-mediated dUTP nick-end labeling (TUNEL) assay (Promega, USA), according to manufacturer’s instructions. For CC3 staining, the appropriate HRP-conjugated secondary antibody (DAKO EnVision, Belgium) was used. The nuclei of the tissue sections were counterstained using Mayer’s hematoxylin (Sigma-Aldrich, USA). Apoptosis was quantified by manually counting CC3- and TUNEL-positive cells in three non-sequential whole tumor sections (*n* = 6/treatment). Sections were scored by two independent operators in a masked way using an upright microscope at ×400 magnification (Olympus CX31, USA). Extensive necrotic areas were excluded from the analysis.

### Statistical Analysis

Results are expressed as mean ± SEM, unless otherwise indicated. Statistical differences between two data sets were analyzed by the unpaired Student’s *t* test (two tailed) with Welch’s correction. One-way ANOVA or two-way repeated-measures ANOVA tests were used for multiple comparisons between groups, followed by Bonferroni correction. Pearson correlation *r* was computed to calculate the correlations between uptake of [^99m^Tc]duramycin and CC3- and TUNEL-positive cells in the tumors (Prism v6.01, GraphPad Software, USA). Statistical significance was set at *p* < 0.05 level.

## Results

### Radiolabeling

**Fig. 1:**
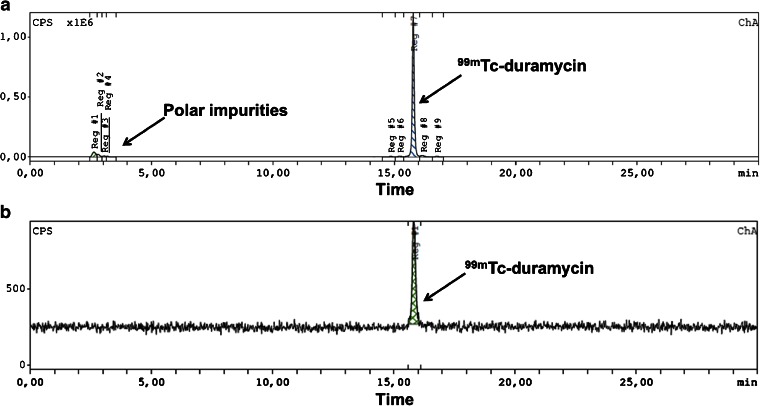
Typical radio-HPLC chromatograms of [^99m^Tc]duramycin **a** before and **b** after purification. *CPS*: counts per second.

Duramycin was labeled with Tc-99m using the HYNIC-tricine-phosphine chelation core. The RCP was 84 ± 6 % (*n* = 11), as assessed by RP-HPLC analysis. As shown in Fig. 1a, after radiolabeling, low amounts of polar impurities could be detected in the [^99m^Tc]duramycin formulation. However, upon HPLC purification, the RCP increased to 99 ± 2 % (*n* = 7; Fig. 1b).

### Dosimetry and Biodistribution Studies

**Fig. 2:**
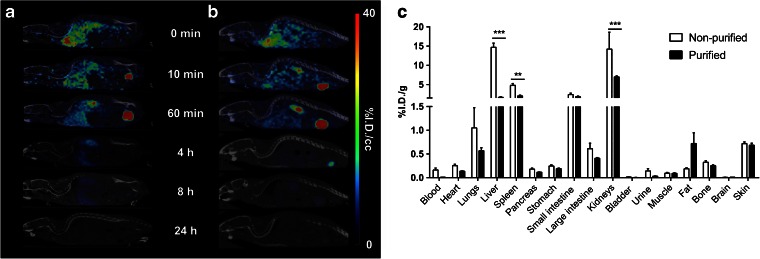
Representative μSPECT-CT images up to 24 h post injection of **a** non-purified and **b** purified 37 MBq [^99m^Tc]duramycin in healthy mice. **c** *Ex vivo* biodistribution (%I.D./g ± SEM) at 24 h p.i. Normalized images were scaled according to the percent injected dose (tissue uptake_[kBq/cc]_/injected dose_[kBq]_*100). Images are presented in the sagittal orientation. **p < 0.01, ***p < 0.001, significantly different from non-purified [^99m^Tc]duramycin.

We then explored the influence of purification on the biodistribution profile of [^99m^Tc]duramycin. [^99m^Tc]duramycin cleared rapidly *via* the kidneys resulting in general low background from 4 h until 24 h p.i. (Fig. 2a, b). Four hours p.i., the purified radiotracer demonstrated faster blood clearance (0.62 ± 0.27 *vs.* 1.46 ± 0.46%I.D./cc for the non-purified radiotracer; *p* = 0.025) and renal excretion (6.82 ± 0.41 *vs.* 8.58 ± 0.15%I.D./cc for the non-purified radiotracer; *p* = 0.010). Furthermore, the non-purified radiotracer showed higher initial accumulation in the liver that remained elevated until 24 h p.i. as evidenced by *ex vivo* biodistribution (Fig. 2c) (14.69 ± 1.09 *vs.* 1.66 ± 0.29%I.D./g for the purified radiotracer; *p* < 0.001). Additionally, non-purified radiotracer showed higher splenic and renal uptake (4.80 ± 0.46; *p* = 0.006 and 14.21 ± 4.39%I.D./g; *p* < 0.001, respectively) when compared with the purified tracer (2.04 ± 0.35 and 6.98 ± 0.42%I.D./g, respectively) (Fig. 2c). Therefore, this intense uptake in non-targeted organs makes it difficult to interpret the activity in surrounding tissues, resulting in images with lower signal-to-background ratio (Fig. 2b). HPLC purification of [^99m^Tc]duramycin resulted in images with lower background levels, enabling vivid tumor imaging. No significant differences in radiotracer uptake could be detected in the remaining organs analyzed 24 h p.i. (Fig. 2c).

The highest level of disintegrations per organ occurred in the liver for the non-purified and in the urinary bladder for the purified radiotracer with residence times of 1.02 h and 6.43 × 10^−1^ h, respectively (Table 1). Table 2 summarizes the mean organ absorbed dose estimates for [^99m^Tc]duramycin. The mean ED were estimated to be 3.74 × 10^−3^ ± 3.43 × 10^−4^ and 3.19 × 10^−3^ ± 2.16 × 10^−4^ mSv/MBq for the non- and purified [^99m^Tc]duramycin, respectively. Interestingly, for the non-purified radiotracer the highest absorbed dose was received by the urinary bladder wall (1.66 × 10^−2^ ± 4.55 × 10^−3^ mGy/MBq), followed by the liver (1.27 × 10^−2^ ± 5.97 × 10^−3^ mGy/MBq). In the case of purified [^99m^Tc]duramycin, the highest absorbed doses were received by the urinary bladder wall (2.70 × 10^−2^ ± 3.05 × 10^−3^ mGy/MBq) and the uterus (4.57 × 10^−3^ ± 3.42 × 10^−4^ mGy/MBq).

**Table 1:** Mean human residence times (in hour) estimates for non-purified and purified [^99m^Tc]duramycin

	Non-purified (*n* = 3) (*f* = 0.519; *t*_1/2_ = 2.950 h)		Purified (*n* = 6) (*f* = 0.697; *t*_1/2_ = 2.151 h)	
Target organs	Mean	SD	Mean	SD
Heart contents	2.44 × 10^−2^	7.52 × 10^−3^	2.70 × 10^−2^	6.15 × 10^−3^
Kidneys	1.30 × 10^−1^	5.73 × 10^−2^	5.49 × 10^−2^	1.30 × 10^−2^
Liver	**1.02**	5.09 × 10^−1^	7.50 × 10^−2^	2.03 × 10^−2^
Urinary bladder contents	3.69 × 10^−1^	1.22 × 10^−1^	**6.43 × 10** ^**−1**^	7.75 × 10^−2^
Remaining body	3.41	6.23 × 10^−1^	2.54	2.13 × 10^−1^

*f* mean fraction leaving the body *via* the urinary excretion system, *t*_*1/2*_ mean biological half-life of the excretion

Highest mean human residence times are presented in bold

**Table 2:** Mean human organ absorbed dose (milligray per megabecquerel) for different organs and effective dose (millisievert per megabecquerel) estimates for non- and purified [^99m^Tc]duramycin

	Non-purified (*n* = 3)		Purified (*n* = 6)	
Organs	Mean	SD	Mean	SD
Adrenals	3.90 × 10^−3^	1.13 × 10^−3^	1.74 × 10^−3^	1.23 × 10^−4^
Brain	1.54 × 10^−3^	2.86 × 10^−4^	1.14 × 10^−3^	9.39 × 10^−4^
Breasts	1.51 × 10^−3^	3.04 × 10^−4^	9.59 × 10^−4^	7.44 × 10^−5^
Gallbladder wall	5.38 × 10^−3^	1.84 × 10^−3^	1.84 × 10^−3^	1.25 × 10^−4^
LLI wall	3.01 × 10^−3^	2.26 × 10^−4^	2.97 × 10^−3^	1.86 × 10^−4^
Small intestine	3.01 × 10^−3^	4.53 × 10^−4^	2.22 × 10^−3^	1.34 × 10^−4^
Stomach wall	2.63 × 10^−3^	5.57 × 10^−4^	1.59 × 10^−3^	1.16 × 10^−4^
ULI wall	3.10 × 10^−3^	5.71 × 10^−4^	2.03 × 10^−3^	1.26 × 10^−4^
Heart wall	3.12 × 10^−3^	7.80 × 10^−4^	1.91 × 10^−3^	1.63 × 10^−4^
Kidneys	8.45 × 10^−3^	3.27 × 10^−3^	3.63 × 10^−3^	6.31 × 10^−4^
Liver	**1.27 × 10** ^**−2**^	5.97 × 10^−3^	1.64 × 10^−3^	2.39 × 10^−4^
Lungs	2.52 × 10^−3^	6.00 × 10^−4^	1.36 × 10^−3^	1.03 × 10^−4^
Muscle	2.12 × 10^−3^	3.35 × 10^−4^	1.55 × 10^−3^	9.64 × 10^−5^
Ovaries	3.15 × 10^−3^	2.59 × 10^−4^	2.96 × 10^−3^	1.78 × 10^−4^
Pancreas	3.79 × 10^−3^	1.02 × 10^−3^	1.82 × 10^−3^	1.30 × 10^−4^
Red marrow	2.16 × 10^−3^	3.76 × 10^−4^	1.48 × 10^−3^	9.73 × 10^−5^
Osteogenic cells	5.51 × 10^−3^	9.74 × 10^−4^	3.92 × 10^−3^	2.91 × 10^−4^
Skin	1.34 × 10^−3^	2.33 × 10^−4^	9.56 × 10^−4^	6.88 × 10^−5^
Spleen	2.48 × 10^−3^	4.93 × 10^−4^	1.59 × 10^−3^	1.17 × 10^−4^
Testes	2.11 × 10^−3^	1.86 × 10^−4^	2.07 × 10^−3^	1.28 × 10^−4^
Thymus	2.03 × 10^−3^	3.93 × 10^−4^	1.39 × 10^−3^	1.07 × 10^−4^
Thyroid	1.87 × 10^−3^	3.42 × 10^−4^	1.38 × 10^−3^	1.13 × 10^−4^
Urinary bladder wall	**1.66 × 10** ^**−2**^	4.55 × 10^−3^	**2.70 × 10** ^**−2**^	3.05 × 10^−3^
Uterus	4.05 × 10^−3^	2.43 × 10^−4^	**4.57 × 10** ^**−3**^	3.42 × 10^−4^
Total body	2.52 × 10^−3^	4.76 × 10^−4^	1.62 × 10^−3^	1.01 × 10^−4^
Effective dose	**3.74 × 10** ^**−3**^	3.43 × 10^−4^	**3.19 × 10** ^**−3**^	2.16 × 10^−4^

Highest mean human organ absorbed doses and effective dose are presented in bold

The data obtained indicate less optimal dosimetry and imaging properties for non-purified [^99m^Tc]duramycin. Therefore, Tc-99m labeled-duramycin was only used after HPLC purification in the following.

### *In Vitro* Uptake

**Fig. 3:**
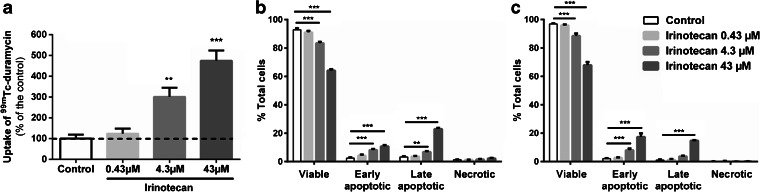
**a**. *In vitro* uptake of [^99m^Tc]duramycin in COLO205 cells after incubation with irinotecan. Detection of apoptosis by **b** Annexin-V and **c** Caspase-3/7 assays. **p < 0.01, ***p < 0.001, significantly different from control.

As shown in Fig. 3a, treatment with 4.3 and 43 μM of chemotherapeutic agent irinotecan significantly increased the binding of the radiotracer to 301.50 ± 43.15 % (*p* = 0.003) and 479.90 ± 49.44 % (*p* < 0.001), respectively, when compared to untreated cells (100.00 ± 19.09 %). Importantly, this marked binding of [^99m^Tc]duramycin by the cells was associated with an increase in apoptosis levels as a result of irinotecan treatment, as demonstrated by the good correlation with the binding of annexin-V and effector caspase activation. Moreover, cell treatment did not result in an increased level of cell necrosis (Fig. 3b, c).

### *In Vivo* Uptake

**Fig. 4:**
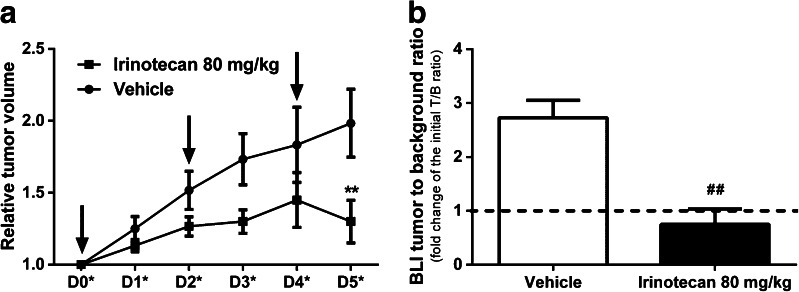
**a** Tumor growth curves for vehicle-treated (*circles*) and irinotecan-treated (*squares*) tumors. The *arrows* indicate the days of treatment administration. **b** BLI signal change after tumor treatment. **p < 0.05 significantly different from the vehicle at D5*; ^##^ *p* < 0.01 significantly different from the vehicle.

**Fig. 5:**
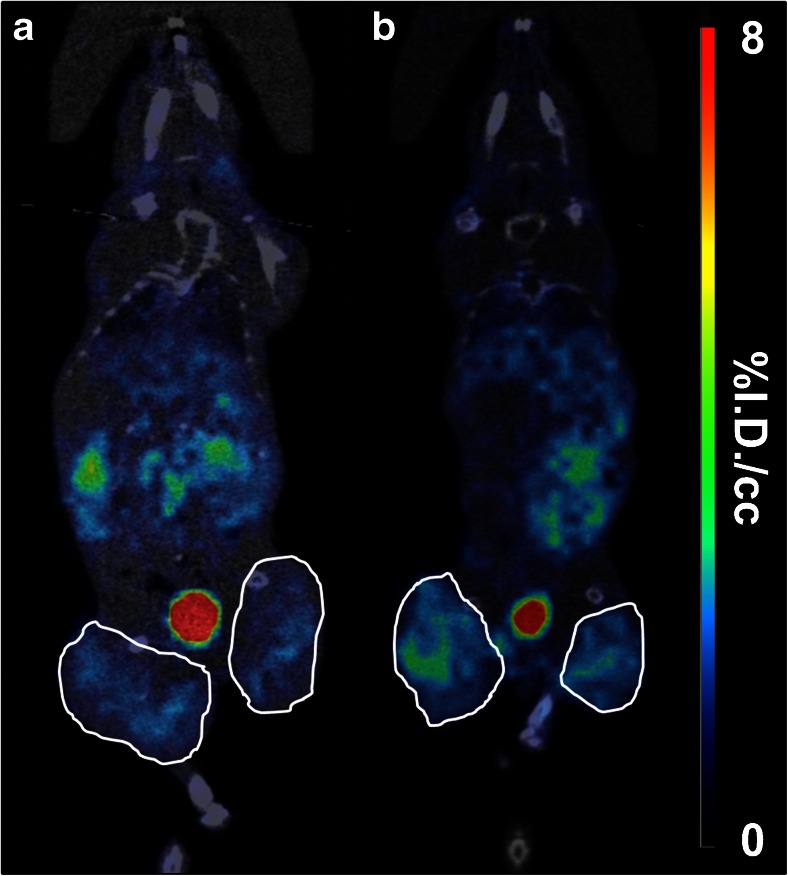
Representative μSPECT-CT images 4 h p.i. of tumor-bearing mice 24 h after the last course of treatment with **a** vehicle and **b** 80 mg/kg irinotecan. Tumors are *encircled*.

COLO205 cells were inoculated in both hind limbs of nude mice in order to reduce the number of animals to be used in this study. Importantly, baseline tumor growth (before treatment) was comparable in both flanks for all the mice (data not shown). The tumor growth curves for each treatment are presented in Fig. 4a. While in vehicle-treated animals the tumors exponentially increased in volume until day 5 (1.98 ± 0.23), in mice treated with irinotecan, tumor growth was significantly inhibited at day 5 (1.30 ± 0.14; *p* = 0.008), which corresponded to a tumor growth inhibition of 34.4 ± 6.9 %. Also, after treatment the bioluminescent signal in the tumors of irinotecan-treated mice was significantly diminished (0.75 ± 0.28; *p* = 0.001) when comparing with the control (Fig. 4b). Although treatment with irinotecan is normally associated with myelosuppression, at the time point used for imaging no significant differences were found in [^99m^Tc]duramycin bone marrow uptake between irinotecan- and vehicle-treated mice (SUV, 0.12 ± 0.02 and 0.11 ± 0.01, respectively; *p* = 0.913). No overall toxicity with respect to body weight loss was observed at the dose of 80 mg/kg irinotecan (data not shown). Based on Fig. 2, we determined that 4 h post [^99m^Tc]duramycin injection is the optimal time point for imaging, allowing enough blood clearance and low background for tumor imaging. Accordingly, Fig. 5 shows μSPECT-CT images 4 h p.i. of tumor-bearing mice 24 h after the last vehicle (Fig. 5a) or irinotecan (Fig. 5b) treatment. The mean tumor uptake value of [^99m^Tc]duramycin was significantly higher in the irinotecan-treated group (148.50 ± 25.42%I.D./g/kg; *p* = 0.008), compared to the vehicle-treated group (40.58 ± 2.81%I.D./g/kg). Similarly tumor-to-muscle and tumor-to-blood uptake ratios for the irinotecan-treated mice were 2-fold (*p* < 0.001) and 6-fold higher (*p* < 0.001) over the vehicle–treated mice, respectively. No correlation was found between tumor size and radiotracer uptake in the tumors (Pearson *r* =−0.29; *p* = 0.36).

### *Ex Vivo* Validation

The uptake of [^99m^Tc]duramycin in the irinotecan-treated tumors (1.65 ± 0.13%I.D./g) was significantly higher than in control tumors (0.74 ± 0.04%I.D./g; *p* < 0.001), as assessed by *γ*-counting. The treatment with irinotecan did not induce differences in radiotracer uptake in the remaining organs when comparing with the control (data not shown).

**Fig. 6:**
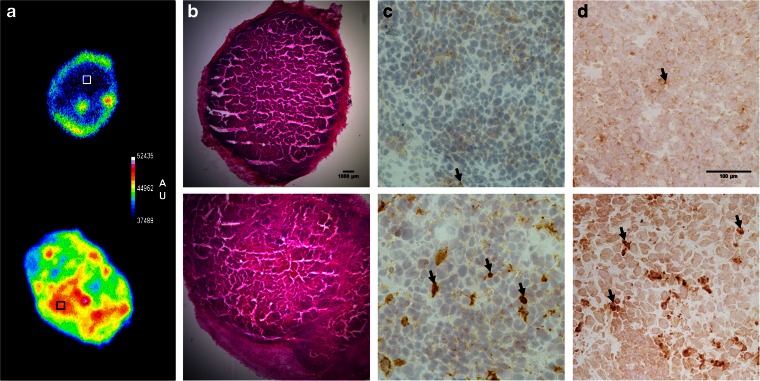
Histological and autoradiography analysis of tumor specimens. **a** Representative autoradiographic images, **b** hematoxylin and eosin staining, **c** cleaved caspase-3 immunostaining, and **d** TUNEL staining for vehicle-treated (*upper panels*) and irinotecan-treated (*lower panels*) tumors. Cells in *brown* were positive for caspase-3 and TUNEL stainings (*arrows*). *Boxes* indicate the areas where zoomed images were acquired. *AU*: arbitrary units.

Figure 6 shows representative autoradiographs and H&E-, CC3-, and TUNEL-stained sections of tumors from mice treated with vehicle and irinotecan. Autoradiographs show a heterogeneous uptake of [^99m^Tc]duramycin in the tumors, which was higher in tumors treated with irinotecan (Fig. 6a). Compared with tumors from vehicle-treated mice, treatment with irinotecan caused cell shrinkage and condensed cytoplasm (Fig. 6b), formation of apoptotic bodies, increase in the number of CC3-positive cells (Fig. 6c; arrows) (9.5 ± 1.1 *vs.* 1.8 ± 0.4 in the control; *p* < 0.001), and DNA fragmentation, as assessed by an increase in the number of TUNEL-positive cells (Fig. 6d; arrows) (10.5 ± 1.6 *vs.* 3.5 ± 0.7 in the control; *p* = 0.006). Most importantly, this extensive apoptotic response was highly correlated with [^99m^Tc]duramycin uptake in the tumors (CC3, *r* = 0.85 and *p* < 0.001, and TUNEL, *r* = 0.92 and *p* < 0.001). The distribution of the apoptotic cells in the tumors matched the distribution of [^99m^Tc]duramycin radioactivity. In control tumors, cold spots colocalized with low number of apoptotic cells, whereas in treated tumors high number of apoptotic cells corresponded to hot spots with high radioactivity (Fig. 6; boxes).

## Discussion

A kit formulation was developed by Zhao and Li to allow a rapid, consistent, and reproducible radiolabeling of duramycin with Tc-99m with high RCP and specific activity [18]. Radiopharmaceutical kits should provide a simplified labeling procedure, yielding high-purity compounds without the need to remove radiochemical impurities. In this study however, we found that the single-step kit formulation resulted in [^99m^Tc]duramycin with unsatisfactory RCP (84 %), hence does not meet the quality specifications desired (>95 % RCP). Based on these results, we conducted a preliminary comparative study on the impact of column purification in animal imaging experiments. Purification of the radiopharmaceutical will remove other radiolabeled species and excess “unlabeled” HYNIC-duramycin that may compete with the radiopharmaceutical in normal or tumor tissues. Our study provides evidence that purification of the [^99m^Tc]duramycin has a prominent impact on the biodistribution profile in mice. Indeed, injection of purified [^99m^Tc]duramycin led to rapid washout of activity from the blood pool and fast elimination primarily *via* renal clearance, as previously described in rats [16]. On the contrary, the non-purified radiotracer showed a slower blood clearance, high spleen uptake, and a combination of urinary and hepatobiliary clearance. A possible present impurity is Tc-99m tin colloid resulting from oxidation and hydrolysis of tin during the labeling [25]. It is well described that tin colloids result in high liver and spleen uptake [26] due to phagocytosis by the reticuloendothelial cells in these organs. However, since a similar biodistribution profile was obtained using [^18^F]duramycin [27], colloid formation cannot exclusively explain the high liver and spleen uptake for non-purified [^99m^Tc]duramycin. Another possible explanation for the higher blood pool activity and liver uptake of non-purified [^99m^Tc]duramycin might be the competition of present unlabeled HYNIC-duramycin and co-ligands with the tracer in the excretion pathway. Also, the abundant expression of PE in lipoproteins in the liver of mice [28] may explain the increased liver uptake of [^99m^Tc]duramycin. Towards the use of [^99m^Tc]duramycin in a mouse model of cancer, it is highly desirable that this radiotracer clears from non-target organs, such as liver and spleen, so that a high tumor-to-background ratio can be achieved. Future efforts will be directed towards optimization of the kit composition, radiolabeling conditions, and purification methodologies (*e.g.*, solid-phase extraction), in order to obtain injection-ready [^99m^Tc]duramycin sufficiently amenable for clinical use.

Since the dosimetry profile of [^99m^Tc]duramycin has not yet been described, we extrapolated the human organ absorbed radiation doses from calculated residence times in mouse organs obtained by whole-body μSPECT-CT, as useful information towards the clinical translation of [^99m^Tc]duramycin. Notably, for both non-purified (0.0037 mSv/MBq) as purified (0.0032 mSv/MBq) [^99m^Tc]duramycin the mean ED were lower than for other apoptosis imaging agents such as [^18^F]ICMT11 (0.025 mSv/MBq) [29], [^18^F]CP18 (0.0056 mSv/MBq) [30] or [^99m^Tc]HYNIC-annexin V (0.011 mSv/MBq) [31]. Moreover, this dosimetry study showed that [^99m^Tc]duramycin meets the criteria for radiation exposure towards future use of this tracer in a clinical set-up. The estimated radiation doses are within the limits described by the European guidelines [32] and US Food and Drug Administration [33]. Importantly, frequent bladder voiding in a clinical imaging scenario, would decrease the absorbed dose to the urinary bladder wall, which was the critical organ receiving the highest dose using the non-purified (1.66 × 10^−2^ mGy/MBq) and purified (2.70 × 10^−2^ mGy/MBq) [^99m^Tc]duramycin. Another organ receiving a relatively high dose using the non-purified radiotracer was the liver (1.27 × 10^−2^ mGy/MBq), reflecting slower excretion kinetics and higher radiotracer retention in the liver, compared to the purified radiotracer. Taken together, these findings suggest that HPLC-purified [^99m^Tc]duramycin is the better candidate for prospective human studies.

Molecular imaging of cell death has gained interest in oncology for monitoring tumor response to cancer therapy. Radiolabeled annexin-V was the first cell death-specific probe to be evaluated clinically [31]. The exposure of membrane aminophospholipids such as PS and PE in the extracellular milieu provides surrogate markers for detecting cell death. PE presents one of the most abundant targets in the membrane bilayer, only preceded by phosphatidylcholine. This higher availability of binding sites represents a clear advantage of duramycin over PS-binding counterparts (*e.g.*, annexin-V). Other limitations of annexin-V include limited tumor penetration [34] and slow clearance from non-targeted tissues [10] due to its large protein structure and high MW (31–36 kDa), reducing signal-to-background ratios. The small (∼2 kDa) polypeptide structure of duramycin enables better tissue penetration and fast clearance from non-targeted organs, which renders it a good candidate biomarker for molecular imaging of cell death in oncology. Besides aminophospholipid targeting tracers other molecules targeting additional hallmarks of apoptosis have been developed. Most notably, [^18^F]ICMT-11 is a non-peptide isatin sulfonamide with nanomolar binding affinity for caspase-3/7 [29]. Less attractive in this family of molecules is the potential suboptimal metabolic stability, in part due to hydroxylation at the isatin aromatic ring [35] and non-specific interactions of the dicarbonyl functionality at the cysteine binding site of the enzyme [36], which may result in unsatisfactory PET imaging. In contrast, duramycin has unique characteristics that confer this radiotracer a superior metabolic stability. The four covalent intramolecular bridges and the absence of a free peptide terminus confer this peptide a better stability and resistance to *in vivo* degradation [16]. We examined the metabolic stability of [^99m^Tc]duramycin in normal CD1−/− mice. According to the radio-HPLC analysis of plasma samples, the majority of the injected [^99m^Tc]duramycin remains unchanged after 4 and 24 h p.i. (see Online Resource). Recently another interesting PET radiotracer, [^18^F]ML-10 has been described, targeting a complex set of features of apoptotic cells [37]. This tracer showed to be a promising tool for molecular imaging of apoptosis and is currently in Phase I/II clinical studies. Although PET radiotracers benefit from the superior sensitivity of PET, SPECT imaging has the advantage of having lower operational costs since it does not require an on-site cyclotron and Tc-99m generators are installed in most hospitals, making its use more suitable in clinical routine.

Above-mentioned factors account for [^99m^Tc]duramyin’s suitability for imaging of cell death. Although the cell death imaging properties of this tracer have been previously explored in animal models of cerebral [17] and myocardial [18] infarction, pulmonary hyperoxia [38], and whole-body irradiation [20], to our knowledge, imaging of treatment response to cancer therapy using [^99m^Tc]duramycin has not yet been described. We therefore explored the cell death targeting properties of [^99m^Tc]duramycin in a mouse model of colorectal cancer treated with the chemotherapy drug irinotecan. This chemotherapy treatment was chosen since campothecin–analog irinotecan is a key component of first- and second-line clinical therapy regimens for colorectal cancer [39], which has already shown a cytotoxic effect in COLO205 cells [8]. SPECT imaging with [^99m^Tc]duramycin revealed an increased accumulation of the radiotracer in the tumors 24 h after the last irinotecan treatment. Additionally, the treatment induced tumor regression, as assessed by caliper measurements, and extensive apoptosis, as evidenced by cleaved caspase-3 and TUNEL stainings. Similarly to other phospholipid-binding imaging agents, [^99m^Tc]duramycin may not bind exclusively to apoptotic cells because necrotic tumor areas also have numerous accessible PE. However, as the COLO205 tumors lacked wide-spread regions of necrosis, as observed macroscopically during sectioning and microscopically by H&E staining (Fig. 6b), the strong correlation between the number of apoptotic cells and tracer tumor uptake *in vivo* suggests that apoptotic tumor cells largely contributed to the enhanced radiotracer uptake in treated tumors. Moreover, *in vitro* [^99m^Tc]duramycin uptake in irinotecan treated COLO205 cells occurred in parallel to annexin-V binding and effector caspase activation, indicating exposure of PS on the cell membrane surface and caspase-akin selective uptake into cells undergoing apoptosis, while membrane integrity is still preserved, as evidenced by the exclusion of 7-amino-actinomycin D (necrosis marker).

It is possible that binding to PE in the tumors may be accompanied by nonspecific uptake. However, we found no evidence of this possibility, since radiotracer uptake was not increased in the tumors of vehicle-treated mice, suggesting a specific treatment-induced uptake in the tumors. This specificity of [^99m^Tc]duramycin for cell death has previously been reported, by Zhao and colleagues [16]. Using inactivated duramycin they demonstrated a PE dependent uptake of [^99m^Tc]duramycin in infarcted tissue [16]. To further evaluate non-specific binding of the tracer in treated tumors a blocking study and imaging study with inactivated duramycin will be performed. Furthermore, the use of [^99m^Tc]duramycin will also be validated using other types of therapy (*e.g.*, radiotherapy and targeted therapy) and other preclinical tumor models with the aim of future SPECT imaging in humans.

## Conclusion

Our results show that [^99m^Tc]duramycin has favorable dosimetry estimates and specifically accumulates in apoptotic cells of tumors responding to chemotherapy, supporting [^99m^Tc]duramycin as a potential candidate for the assessment of individualized responses to conventional therapy in cancer patients.

## Electronic Supplementary Material

ESM 1 (PDF 145 kb)

## References

[1] Therasse P, Arbuck SG, Eisenhauer EA (2000). New guidelines to evaluate the response to treatment in solid tumors. European Organization for Research and Treatment of Cancer, National Cancer Institute of the United States, National Cancer Institute of Canada. J Natl Cancer Inst.

[2] Norbury CJ, Zhivotovsky B (2004). DNA damage-induced apoptosis. Oncogene.

[3] Hanahan D, Weinberg RA (2011). Hallmarks of cancer: the next generation. Cell.

[4] Meiler J, Schuler M (2006). Therapeutic targeting of apoptotic pathways in cancer. Curr Drug Targets.

[5] Amaravadi RK, Thompson CB (2007). The roles of therapy-induced autophagy and necrosis in cancer treatment. Clin Cancer Res.

[6] de Bruin EC, Medema JP (2008). Apoptosis and non-apoptotic deaths in cancer development and treatment response. Cancer Treat Rev.

[7] Belhocine TZ, Prato FS (2011). Transbilayer phospholipids molecular imaging. EJNMMI Res.

[8] Vangestel C, Van de Wiele C, Van Damme N (2011). (99)mTc-(CO)(3) His-annexin A5 micro-SPECT demonstrates increased cell death by irinotecan during the vascular normalization window caused by bevacizumab. J Nucl Med.

[9] Vangestel C, Van de Wiele C, Mees G (2012). Single-photon emission computed tomographic imaging of the early time course of therapy-induced cell death using technetium 99m tricarbonyl His-annexin A5 in a colorectal cancer xenograft model. Mol Imaging.

[10] Wen X, Wu QP, Ke S (2003). Improved radiolabeling of PEGylated protein: PEGylated annexin V for noninvasive imaging of tumor apoptosis. Cancer Biother Radiopharm.

[11] Wang F, Fang W, Zhang MR (2011). Evaluation of chemotherapy response in VX2 rabbit lung cancer with ^18^F-labeled C2A domain of synaptotagmin I. J Nucl Med.

[12] Wyffels L, Gray BD, Barber C (2011). Synthesis and preliminary evaluation of radiolabeled bis(zinc(II)-dipicolylamine) coordination complexes as cell death imaging agents. Bioorg Med Chem.

[13] Neves AA, Brindle KM (2014). Imaging cell death. J Nucl Med.

[14] Callahan MK, Williamson P, Schlegel RA (2000). Surface expression of phosphatidylserine on macrophages is required for phagocytosis of apoptotic thymocytes. Cell Death Differ.

[15] Zhao M (2011). Lantibiotics as probes for phosphatidylethanolamine. Amino Acids.

[16] Zhao M, Li Z, Bugenhagen S (2008). ^99m^Tc-labeled duramycin as a novel phosphatidylethanolamine-binding molecular probe. J Nucl Med.

[17] Zhang Y, Stevenson GD, Barber C (2013). Imaging of rat cerebral ischemia-reperfusion injury using (99m)Tc-labeled duramycin. Nucl Med Biol.

[18] Zhao M, Li Z (2012). A single-step kit formulation for the (99m)Tc-labeling of HYNIC-Duramycin. Nucl Med Biol.

[19] Wang L, Wang F, Fang W (2015). The feasibility of imaging myocardial ischemic/reperfusion injury using (99m)Tc-labeled duramycin in a porcine model. Nucl Med Biol.

[20] Johnson SE, Li Z, Liu Y, Moulder JE, Zhao M (2013). Whole-body imaging of high-dose ionizing irradiation-induced tissue injuries using ^99m^Tc-duramycin. J Nucl Med.

[21] Kirschner AS, Ice RD, Beierwaltes WH (1975). Radiation dosimetry of 131I-19-iodocholesterol: the pitfalls of using tissue concentration data—reply. J Nucl Med.

[22] Stabin MG, Sparks RB, Crowe E (2005). OLINDA/EXM: the second-generation personal computer software for internal dose assessment in nuclear medicine. J Nucl Med.

[23] (1991) 1990 Recommendations of the International Commission on Radiological Protection. Ann ICRP 21:1-201 2053748

[24] Cloutier RJ, Smith SA, Watson EE (1973). Dose to the fetus from radionuclides in the bladder. Health Phys.

[25] Spies H, Pietzsch HJ (2007) Stannous chloride in the preparation of ^99m^Tc pharmaceuticals. In: Zolle I (ed) Technetium-99m pharmaceuticals: preparation and quality control in nuclear medicine. Springer, Berlin Heidelberg, pp 59–66

[26] Shim KM, Kim SE, Moon C (2009). A detailed examination of pulmonary uptake of (99m)Tc-Tin colloid in healthy mature miniature pigs. In Vivo.

[27] Yao S, Hu K, Tang G (2014). Positron emission tomography imaging of cell death with [(18)F]FPDuramycin. Apoptosis.

[28] Vance JE (2008). Phosphatidylserine and phosphatidylethanolamine in mammalian cells: two metabolically related aminophospholipids. J Lipid Res.

[29] Challapalli A, Kenny LM, Hallett WA (2013). ^18^F-ICMT-11, a caspase-3-specific PET tracer for apoptosis: biodistribution and radiation dosimetry. J Nucl Med.

[30] Doss M, Kolb HC, Walsh JC (2013). Biodistribution and radiation dosimetry of ^18^F-CP-18, a potential apoptosis imaging agent, as determined from PET/CT scans in healthy volunteers. J Nucl Med.

[31] Kemerink GJ, Liu X, Kieffer D (2003). Safety, biodistribution, and dosimetry of ^99m^Tc-HYNIC-annexin V, a novel human recombinant annexin V for human application. J Nucl Med.

[32] Verbruggen A, Coenen HH, Deverre JR (2008). Guideline to regulations for radiopharmaceuticals in early phase clinical trials in the EU. Eur J Nucl Med Mol Imaging.

[33] Food and Drug Administration (2011) Title 21 CFR 361.1 radioactive drugs for certain research uses. National Archives and Records Administration, Office of the Federal Register, Washington DC, pp 325–330

[34] Beekman CA, Buckle T, van Leeuwen AC (2011). Questioning the value of (99m)Tc-HYNIC-annexin V based response monitoring after docetaxel treatment in a mouse model for hereditary breast cancer. Appl Radiat Isot.

[35] Baumann A, Faust A, Law MP (2011). Metabolite identification of a radiotracer by electrochemistry coupled to liquid chromatography with mass spectrometric and radioactivity detection. Anal Chem.

[36] Lee D, Long SA, Murray JH (2001). Potent and selective nonpeptide inhibitors of caspases 3 and 7. J Med Chem.

[37] Cohen A, Shirvan A, Levin G (2009). From the Gla domain to a novel small-molecule detector of apoptosis. Cell Res.

[38] Audi SH, Jacobs ER, Zhao M (2015). *In vivo* detection of hyperoxia-induced pulmonary endothelial cell death using (99m)Tc-Duramycin. Nucl Med Biol.

[39] Fuchs C, Mitchell EP, Hoff PM (2006). Irinotecan in the treatment of colorectal cancer. Cancer Treat Rev.

