# Supplementary material for: Characterization of [99mTc]Duramycin as a SPECT Imaging Agent for Early Assessment of Tumor Apoptosis
Source: Mol Imaging Biol. 2015 Apr 21;17(6):838–47. doi: 10.1007/s11307-015-0852-6 (PMC4641155; doi:10.1007/s11307-015-0852-6)
Supplement: Supplementary file 1 — (PDF 145 kb) [file 11307_2015_852_MOESM1_ESM.pdf]

Characterization of [^{99m}Tc]Duramycin as a SPECT Imaging Agent for Early Assessment of Tumour Apoptosis

Journal: Molecular Imaging and Biology

Filipe Elvas[1,2], Christel Vangestel[1,2], Sara Rapic[1], Jeroen Verhaeghe[1], Brian Gray[3], Koon Pak[3], Sigrid Stroobants[1,2], Steven Staelens[1], Leonie wyffels[1,2]

[1]Molecular Imaging Center Antwerp, University of Antwerp, Antwerp, Belgium; [2]University Hospital Antwerp, Department of Nuclear Medicine, Edegem, Belgium; [3]Molecular Targeting Technologies, Inc., Pennsylvania, USA

For correspondence or reprints contact: Leonie wyffels, Department of Nuclear Medicine, University Hospital Antwerp, Wilrijkstraat 10, 2650 Edegem, Belgium.

Email: leonie.wyffels@uza.be

Telephone number: +3238215699

Fax number: +3238253308

Manuscript category: Original Article

Running Head: Imaging Apoptosis Using [^{99m}Tc]Duramycin

Supplementary Material: ESM

Materials and methods

In vivo metabolite analysis

In vivo plasma stability of [^{99m}Tc]duramycin was evaluated as previously described [1]. Briefly, mice were i.v. injected with 37 MBq purified [^{99m}Tc]duramycin. Four and 24 h p.i. (n=3 for each time point) mixed blood was drawn by cardiac puncture, and the mice were euthanized by cervical dislocation. The blood was collected in EDTA-coated tubes and the plasma fraction was obtained by centrifugation (4000 g for 7 min). The plasma (200 µl) was mixed with an equal amount of cold acetonitrile to enable sample deproteination and counted for radioactivity using an automatic γ-counter. After vortexing and centrifuging for 4 min at 4000 g, the supernatant was separated from the pellet and both fractions were γ-counted to calculate the amount of radioactivity extracted in acetonitrile. To study radiometabolite formation, 100 µl of the supernatant were analyzed by RP-HPLC using the method described before. Eluate fractions of 0.5 min were collected and counted for radioactivity in the γ-counter.

Results

In vivo metabolite analysis

Adult normal CD1-/- nude mice were used to examine the metabolic stability of [^{99m}Tc]duramycin. According to the radio-HPLC analysis of the plasma samples, the majority of the injected radiotracer remains unchanged after 4 and 24 h p.i. (Figs. 1b and c, respectively). One minor polar impurity was detected eluting at 3 min. As it was also visible in the reference radiochromatogram (Fig. 1a), it was not related to in vivo metabolisation of [^{99m}Tc]duramycin. Radiometabolites eluting with a longer retention time (18.5 – 30 min) could also be detected.

Their presence might be the result of a complex formation between [^{99m}Tc]duramycin and blood lipoproteins, which are known to contain PE [2].

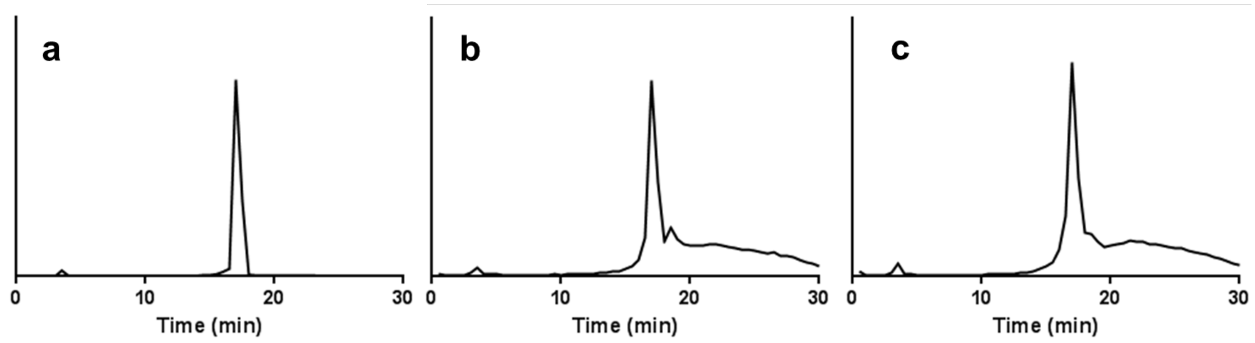

Fig. 1. Representative radio-HPLC chromatograms of [^{99m}Tc]duramycin reference (a), plasma at 4 h (b) and 24 h (c) after injection

References

1. Wyffels L, Thomae D, Waldron AM, Fissers J, Dedeurwaerdere S, Van der Veken P et al. In vivo evaluation of (18)F-labeled TCO for pre-targeted PET imaging in the brain. Nuclear medicine and biology. 2014;41(6):513-23. doi:10.1016/j.nucmedbio.2014.03.023.

2. Zhao M, Li Z, Bugenhagen S. ^{99m}Tc-labeled duramycin as a novel phosphatidylethanolamine-binding molecular probe. Journal of nuclear medicine : official publication, Society of Nuclear Medicine. 2008;49(8):1345-52. doi:10.2967/jnumed.107.048603.
